# Annotations

**Published:** 1905-06-10

**Authors:** 


### ANNOTATIONS.

### Serious Increase of Beri-beri.

The increasing prevalence of beri-beri among the Chinese in Hongkong was referred to some time back in our columns by a contributor who recently visited the Tung Wah Hospital. From the report of the Principal Civil Medical Officer for 1904, which is now before us, we learn that the position is regarded in the colony as serious. Whereas the total admissions in 1903 to the Tung Wah Hospital of persons suffering from beri-beri was 277, in 1904 they rose to 742, the number of fatal cases being no fewer than 329. Moreover, a similar condition of affairs obtains at the Government Civil Hospital in the colony, the cases in 1904 being 70 as against 36 in 1903. In these circumstances, it has very properly been determined by the Hongkong Government to institute a searching inquiry by Dr. Koch, one of the medical staff, and the Government bacteriologist, Dr. Hunter, with the object of ascertaining the causes of the malady. It is hoped that as a result of their researches some light may be thrown upon the disease, about whose etiology so little has yet been discovered. Not only Europeans, but apparently Eurasians also, are exempt from it, and the general opinion is that the mode of living and clothing, or the dietary of the natives, in the countries where it prevails is at the bottom of the mischief. The investigation which it has been decided to hold in Hongkong is of special importance to China, Japan, the Netherlands, Indies, and the Malay Peninsula. We believe that beri-beri is also rife in Siam and Indo-China. At all events, alike in the interests of humanity and of medical science, it is desirable that no opportunity should be lost of securing any information which is likely to render it less difficult to arrest its extension and to treat it successfully.

### Violence in Relation to Coroners' Inquests.

The fact that violence or injury has been one of the events more or less immediately precedent to death is a circumstance which may make an inquest a necessity, and certainly, even though a natural cause for death is obvious, ought to be communicated to the coroner. Medical practitioners sometimes forget this, and by so doing cause trouble both to themselves and to others. Even though the violence is remote and to a moral certainty has no share in the responsibility for the fatal event, its occurrence, and the legal possibilities arising therefrom, ought to be recognised. It is, according to law, the duty of the coroner to hold an inquest in any case in which there is reason to suspect that any person has died a violent or unnatural death, and apparently it lies with the coroner to decide what is sufficient to justify "reasonable suspicion." As a strict matter of right all that the practitioner has to do is either to grant or to refuse a certificate of death, but he will best serve the public interests by putting all the facts before the authorities and accepting their guidance in any doubtful case. Violence, either accidental or otherwise, which, whether in point of time or other circumstances has even an apparent association with the fact of death, ought to be plainly stated, either on the certificate of death or in some other method. A somewhat novel point in the relationship of inquests to deaths possibly related to some violent cause has recently been raised in the Divisional Court in the shape of an application to compel a coroner to hold a second inquiry on the ground that the first had not been complete. The case was that of a man who died in prison to which he had been committed for an assault, and the jury found that death was a result of injuries to the skull received prior to his admission to prison. It is now contended that the inquiry should have endeavoured to determine how and where and why the deceased received the injuries from which he died, and that the coroner should have secured evidence to show who was responsible for the violence that led to the fatal issue. The decision of the Court will be of considerable interest as helping to determine whether the coroner's inquiry is limited to the immediate cause of death or whether a further and absolute inquiry arising out of this must necessarily be instituted.

### Verminous Children.

The Medical Officer of Health for Marylebone has recently presented a report to the Borough Council in regard to the treatment of verminous persons under the "Cleansing of Persons Act." Baths for this purpose were first established in the borough in 1898, and have since that date been used by no fewer than 32,500 persons, including 1,796 females. On the basis of this experience it is concluded that successful application of the provisions of the Act is most likely to be secured by dissociating the baths from the Poor-law administration and by not worrying applicants with a number of unnecessary restrictions. A new departure is now suggested in the direction of making such baths available for cleansing school children affected with pediculi. That children attending school under the compulsion of the law should not run the risk of contamination with vermin seems an obvious claim, and it is so far satisfactory to know that the educational authorities have taken steps to detect infested children and to exclude them from school until they have been properly cleansed. The need for this is rendered obvious by the figures quoted from ten of the public schools in Marylebone, which show that out of 1,378 children examined no less than 297, that is, roughly, 21·5 per cent., suffered from the presence of pediculosis of the scalp. In the borough of St. Pancras the local authorities have used the baths provided under the Act above-mentioned for the cleansing of such children, and the experiment has been so successful that Marylebone has determined to adopt a similar course. The plan includes the issue to the teachers in the elementary schools of a circular announcing the arrangements provided by the Borough Council, and it rests with the teachers to acquaint the parents concerned with the facilities provided. The policy of compulsory education has indeed raised a host of unexpected problems, but even the most ardent opponent of the beginnings of socialism will hardly object to measures necessary to protect children from contamination by lice.

